# CAR-T Cell Therapies in B-Cell Acute Lymphoblastic Leukemia: Emerging Data and Open Issues

**DOI:** 10.3390/cancers17183027

**Published:** 2025-09-16

**Authors:** Caterina Alati, Martina Pitea, Matteo Molica, Luca Scalise, Gaetana Porto, Erica Bilardi, Giuseppe Lazzaro, Maria Caterina Micò, Marta Pugliese, Filippo Antonio Canale, Barbara Loteta, Virginia Naso, Giorgia Policastro, Giovanna Utano, Andrea Rizzuto, Violetta Marafioti, Marco Rossi, Massimo Martino

**Affiliations:** 1 Hematology and Stem Cell Transplantation and Cellular Therapies Unit (CTMO), Department of Hemato-Oncology and Radiotherapy, Grande Ospedale Metropolitano “Bianchi-Melacrino-Morelli”, 89133 Reggio Calabria, Italydr.massimomartino@gmail.com (M.M.); 2 Stem Cell Transplant Program CIC587, 89133 Reggio Calabria, Italy; 3 Department of Hematology-Oncology, Azienda Universitaria Ospedaliera Renato Dulbecco, 88100 Catanzaro, Italylucascalise@yahoo.it (L.S.); rossim@unicz.it (M.R.)

**Keywords:** acute lymphoblastic leukemia, CD19-directed chimeric antigen receptor T-cell, allogeneic CAR-T, CAR-T as consolidation, gene-editing methods

## Abstract

CAR-T therapy has transformed the treatment of relapsed or refractory B-cell acute lymphoblastic leukemia (B-ALL), particularly in pediatric and young adult patients. Many studies report one-year overall survival rates of between 60% and 80% following therapy. Event-free survival rates at one year are around 50–70%, with 40–50% of patients in remission after two years. Despite these impressive results, disease relapse remains a problem. Future CAR-T cell platforms should target multiple antigens, and the optimal design of such constructs must be determined. Modern trials should explore the role of CAR-T cell therapy as a consolidation treatment for patients with high-risk ALL, including those with persistent minimal residual disease at the end of induction/consolidation therapy, an IKZF1-positive gene expression profile, or a TP53 mutation or Ph-like gene expression profile. Improving the efficiency of gene-editing methods could lead to higher success rates in creating CAR-T cells, as well as reducing manufacturing time and costs. Producing universal CAR-T cells from healthy donors could significantly reduce production time and costs. These issues underscore the dynamic and evolving nature of B-ALL research. Ongoing studies and clinical trials are addressing many of these challenges in order to improve outcomes for B-ALL patients and expand the applications of CAR-T cell therapy.

## Simple Summary

CAR-T is effective in treating relapsed or refractory B-cell acute lymphoblastic leukemia. Nevertheless, several challenges exist, such as relapse, severe toxicities, high costs, and limited accessibility. The future promises transformation through engineered CARs targeting multiple antigens, safer and longer-lasting T cells, universal off-the-shelf products, combination therapies, and sophisticated manufacturing. These innovations aim to improve the safety, efficacy, and accessibility of CAR-Tcell therapy.

## 1. Introduction

Acute lymphoblastic leukemia (ALL) is the most common childhood cancer, peaking in incidence between the ages of two and five [1]. In adults, incidence is lower, but it increases with age, peaking in the elderly. Among children, B-ALL constitutes 90% of cases, whereas in adult patients, subtypes of B-ALL represent 75% of cases, including mature B-ALL, which constitutes 5% of adult ALL. The remaining 20% comprises T-cell lineage ALL [2,3].

The treatment landscape for ALL has evolved, with distinct approaches for pediatric and adult populations. Children have a highly favorable prognosis and achieve cure rates of around 85–90% with modern multi-agent chemotherapy. Success rates are lower in adults but are improving due to targeted therapies and transplant strategies. However, approximately 10% of patients with B-ALL do not respond to initial therapy, and 30% to 60% will relapse after first-line chemotherapy [4,5,6,7,8,9]. The prognosis for relapsed/refractory (R/R) patients is poor, and response rates decrease with each subsequent line of salvage therapy. Allogeneic stem cell transplantation (allo-SCT) remains the only curative strategy for patients who have achieved complete remission (CR) through salvage therapy, although the allogeneic procedure is still associated with a high risk of morbidity and mortality [10,11,12,13,14,15,16,17].

Recently, CAR-T has transformed the treatment of relapsed/refractory (R/R) B-ALL [17,18,19]. Table 1 summarizes the various steps involved in CAR-T therapy. One of the first CAR-T constructs investigated was 19-28z, which links the CD19 binding receptor to the costimulatory protein CD28 [20,21]. Other CD19-targeted constructs have been investigated; some comprise an alternative costimulatory protein (4-1BB) and have shown similar results to 19-28z CAR-T [22]. CD22 serves as an important alternative target, especially for CD19-negative relapses. CD22 is expressed on most B-ALL cases and can be targeted when CD19 expression is lost after CD19 CAR T therapy. Advancements in CAR-T design beyond CD19 represent an ongoing therapeutic evolution [23,24]. CD20 is being explored, though expression is more variable in B-ALL compared to mature B-cell lymphomas. BCMA (CD269) shows promise for certain B-ALL subtypes, particularly those with plasma cell features. CD123 (IL-3 receptor alpha) is expressed on some B-ALL blasts and is being investigated as a target. CD10 (CALLA) is highly expressed on many B-ALL cases and represents an attractive target under development.

This paper summarizes results regarding approved and emerging CAR-T and sequential strategies involving allo-SCT in R/R B-ALL. It also expresses an expert opinion, highlighting the outstanding issues and possible future scenarios.

## 2. CAR-T: Pivotal Clinical Trials

Table 2 summarizes the approved therapies for treating R/R B-ALL. Tisagenlecleucel (Tisa-Cel), a genetically modified autologous cell-based product, has shown promising results [25]. The ELIANA trial, a phase II study evaluating Tisa-Cel in 97 pediatric and young adult patients, demonstrated an overall remission rate (ORR) of 81% within three months of infusion, and all patients were MRD-negative [26]. Tisa-Cel has received approval from both the U.S. Food and Drug Administration (FDA) and the European Medicines Agency (EMA) for the treatment of patients <26 years of age with R/R B-ALL. A key aspect of the trial was the long-term follow-up, showing at 36 months a probability of duration of response (DOR), event-free survival (EFS), and overall survival (OS) of 52.2% (36.9–65.5), 44.4% (31.3–56.8), and 62.8% (50.7–72.7), respectively [27]. The manageable adverse events associated with Tisa-Cel further instill confidence in its safety profile, making it a reliable option in the current medical advancements in the treatment of R/R B-ALL.

The efficacy of Tisa-Cel was recently confirmed in a real-life retrospective analysis of 255 patients [28]. With a median follow-up of 13.4 months, the CIBMTR registry data showed a morphologic CR in 85.5% of patients, and MRD negativity was reported in 99% of patients who had achieved a CR. Twelve-month DOR, EFS, and OS rates were 60.9%, 52.4%, and 77.2%, respectively. These outcomes were comparable to those of patients treated in the ELIANA trial.

Brexucabtageneautoleucel (Brexu-Cel) is a genetically modified autologous cell-based product containing T cells transduced ex vivo using a retroviral vector expressing an anti-CD19 CAR comprising a murine anti-CD19 single-chain variable fragment (scFv) linked to a CD28 costimulatory domain and CD3-zeta signaling domain [27]. The ZUMA-3 study evaluated the effectiveness of Brexu-Cel in 78 adult patients with relapsed or refractory B-LLA [29]. The experimental arm showed a CR with an incomplete hematologic recovery (CRi) rate of 73% (CR rate of 60%), a testament to the high remission rates of Brexu-Cel, and a median OS of 25.4 months. Shah and colleagues reported the outcomes after a median follow-up of more than 3 years. Median OS was 25.6 months and was 38.9 months for responders’ patients (n = 58), with nine patients in ongoing remission without subsequent therapies [30]. Patients in ZUMA-3 continued to experience a survival benefit with a 40% 5-year OS rate. Responders had the most significant benefit with a median OS of >5 years (CR/CRi), which was not reached in those with CR. Patients benefited regardless of age, prior therapy, or subsequent allo-SCT status; however, small subgroups and unbalanced patient characteristics limit the interpretation of post hoc subgroup analyses. No new safety signals were observed, providing reassurance about the effectiveness of Brexu-Cel. FDA and EMA approved Brexu-Cel for adult patients with R/R B-ALL [31].

At the last European Hematology Association (EHA) meeting, Oluwole et al. presented the 5-year follow-up data of ZUMA-3 [32]. Patients continued to experience a survival benefit with a 5-year OS rate of 40%. Responders had the most significant treatment benefit with a median OS of more than 5 years. The OS benefit was consistent across subgroups regardless of age, prior therapy, or subsequent allo-SCT. Five-year OS rates among patients with (n = 29) or without (n = 49) a prior allo-SCT were reported at 36% and 42%, respectively. The benefit for those treated with prior blinatumomab or inotuzumab was less pronounced. At the 5-year update, no new safety signals were identified, and no new T-cell malignancies were reported.

Recently, Roloff and colleagues published a retrospective analysis of 189 adults with relapsed or refractory B-ALL treated with Brexu-Cel outside of clinical trials [33]. Treatment regimens included autologous-SCT for 41% of patients, blinatumomab for 59%, and inotuzumab for 48%. Forty-two percent of patients would have been excluded from ZUMA-3 and received Brexu-Cel in morphologic remission. Of those, 27% were MRD-positive/unknown, and 15% were MRD-negative. With a median follow-up of 11.4 months, 151 patients achieved CR, and 79% were MRD-negative. The median time to relapse was 154 days (range: 25–507), and the median PFS and OS were 9.5 months and not reached, respectively. Adverse events of interest were grade 3/4 CRS in 11% of patients and grade 3/4 ICANS in 31%.

On 21 July of this year, the European Commission granted marketing authorization for obecabtageneautoleucel (Obe-Cel), a CAR-T cell-based therapy developed by the biotech Autolus for the treatment of adult patients aged 26 years and older with relapsed or refractory B-ALL. In the United States, Obe-Cel had already been approved by the FDA in November 2024 [34]. Obe-Cel is an autologous anti-CD19 41BB-ζ CAR-T cell therapy. Unlike Tisa-Cel and Brexu-Cel, which use the same high-affinity single-chain variable fragment (scFv) to recognize CD19, OBE-CEL uses a different intermediate-affinity scFv [35]. This scFv is characterized by a rapid dissociation rate, which can reduce side effects and improve CAR-T cell engraftment and persistence.

The efficacy of Obe-Cel was evaluated in the multicenter, open-label, single-arm, phase I/II FELIX clinical trial, which enrolled adults with R/R CD19-positive B-cell ALL. Eligible patients had to have relapsed disease after remission lasting up to 12 months; R/R B-ALL after two or more prior lines of systemic therapy; or R/R disease three or more months after allo-SCT. The results were published in the November 2024 issue of the *New* *England Journal of Medicine* [36]. In the pivotal cohort (Cohort IIA; n = 94), the CR/CRi rate was 76.6% for patients who received at least one Obe-Cel infusion. The median duration of response for all treated patients was 21.2 months. The median EFS was 11.9 months, and the estimated EFS rates at 6 and 12 months were 65.4% and 49.5%, respectively.

Regarding treatment tolerability, 87 out of 127 patients (68.5%) experienced cytokine release syndrome (CRS), with only 2.4% of patients experiencing grade 3 or higher events. The incidence of grade 3 or higher immune effector cell-associated neurotoxicity syndrome (ICANS) was 7.1%. Severe ICANS was predominantly found in patients with a high bone marrow blast count. The primary consideration is that Obe-Cel yielded a high incidence of durable responses, consistent with previous studies using other CAR-T products. Patients with low/intermediate bone marrow disease burden experienced the most significant benefit in terms of duration of response. Unlike other CAR-Ts, Obe-Cel was associated with a very low incidence of grade 3 or higher immune-related toxic effects, which also correlated with tumor burden.

Updated data from the trial, with approximately 3 years of follow-up, demonstrated that 38.4% of responders maintained their remission without consolidation with allo-SCT or other treatments [37]. Investigators conducted a post hoc analysis of the FELIX trial to understand the long-term activity of the agent in different age groups, and data were presented at the 2025 EHA Congress [38]. Minimal differences in objective response rate, EFS, and OS were observed in patients aged <55 years and ≥55 years. This result was also consistent in the age groups of interest (≥26 years and ≥65 years).

## 3. Sequential Use of CAR T and allo-SCT

CAR T-cells can induce durable remissions; however, relapse remains a significant issue [39]. After achieving remission with CAR-T, allo-SCT consolidation is an evolving strategy in R/R B-ALL, especially in patients at high risk of relapse [33,33,40,41,42,43,44,45,46]. The timing of allo-SCT is a critical point and depends on the patient’s recovery and disease status. Evidence is mainly derived from small retrospective clinical trials. The rationale for proceeding to transplant, patient populations, and details of transplant procedures (e.g., conditioning, prophylaxis of graft-versus-host disease [GVHD], or specific outcomes) are heterogeneous and inconsistently described in the literature. Table 3 summarizes the main studies that used a consolidation approach with allo-SCT after CAR-T.

## 4. Dual-Targeting CAR T

Dual-target CAR T-cell therapy represents an exciting advancement in the field of cancer immunotherapy, potentially offering improved efficacy and reduced risk of treatment resistance compared to single-target approaches [47].

CARPAL is an ongoing academic, multicenter, open-label, single-arm phase I trial evaluating dual-targeting CD19 + CD22 CAR-T-cells in patients with R/R B-ALL [48,49]. Patients consist of children and young adults (age < 24 years). The study is evaluating the safety, efficacy, and duration of response. In preliminary analysis of twelve heavily pre-treated patients, six experienced relapses after allo-SCT, and four were previously treated with Tisa-Cel [50]. In patients who received a single dose of 10^6^ CD19 + CD22 CAR-T, the rate of MRD-negative CR at 2 months was 83% (n/N = 10/12); the 12-month rates of OS and EFS were 75% and 60%, respectively. The dual-targeting CD19 + CD22 CAR-T was well tolerated, and 11 out of 12 patients developed CRS (with no grade 3 or higher CRS events); 5 patients received tocilizumab. Five patients had grade 1/2 ICANS, and one patient developed grade 4 neurotoxicity/ICANS.

## 5. Allogeneic CAR-T

The use of allogeneic CAR-T cells from healthy donors has many potential advantages over autologous approaches (see Table 4) [51].

BALLI-01 is an open-label dose-escalation and dose-expansion study to evaluate the safety, expansion, persistence, and clinical activity of UCART22 (Allogeneic Engineered T-cells Expressing Anti-CD22 Chimeric Antigen Receptor) in patients with R/R CD22+ B-ALL [52]. UCART22 has been engineered to minimize the risk of GVHD by disrupting the T-cell receptor alpha constant and CD52 genes. Eligibility criteria consisted of an age range of 15–70 years and CD22 expression of ≥70% [53]. The trial consisted of two manufacturing processes for UCART22: the first was manufactured by a CMO; the second was manufactured in-house. The preliminary results from patients treated with UCART22, manufactured by a CMO, showed that meaningful responses were achieved at the highest dose level, and UCART22 was well tolerated. Lymphodepletion with fludarabine, cyclophosphamide, and alemtuzumab improved UCART22 expansion compared to fludarabine and cyclophosphamide alone [54]. UCART22, manufactured in-house, was evaluated in three patients at ×10^6^ cells/kg after FCA depletion. Responses were observed in 2/3 patients (67%); 2/3 patients (67%) experienced any-grade CRS, with no occurrences of grade 3 or higher CRS. No cases of ICANS were reported. The study is enrolling patients at DL2i (2.5 × 10^6^ cells/kg) with UCART22 manufactured in-house.

RD06-03 is a phase I trial of anti-CD19 CAR T-cell therapy in R/R B-ALL. RD06-03 is an allogeneic anti-CD19 CAR T-cell engineered to overexpress the ANSWER molecule, which enhances persistence and promotes resistance to rejection by host T-cells and NK cells. A phase I dose escalation trial is investigating the safety and efficacy of RD06-03 [55]. Eligibility criteria consist of age 3–70 years; CD19+ R/R Ph- B-ALL; Ph+ permitted if R/R after ≥2 tyrosine kinase inhibitors. In preliminary analysis of six patients, RD06-03 showed clinical efficacy, with five patients achieving MRD-negative CR/Cri. Patients who achieved CR/CRi received ≥0.3 mg/kg CAR T-cells after lymphodepletion with fludarabine plus cyclophosphamide. RD06-03 was well tolerated. Most reported TEAEs were cytopenias; 4/6 patients had grade 1 CRS, with no grade 3 or higher CRS events. No DLT, neurotoxicity, or GVHD was observed.

## 6. Fast CAR-T

Chinese researchers recently created CD19 fast-CAR-T, which can be produced within a single day [56]. They conducted a study in 44 patients with R/R B-ALL to evaluate the effectiveness and safety of fast-CAR-T compared to those of conventional CAR-T. Compared with the conventional, the fast-CAR-T had significantly higher CR and MRD-negative rates (95.7% versus 91.3% and 71.4% versus 66.7%, respectively). No significant differences were observed in the 1-year or 2-year OS and LFS rates between the two arms. The incidence of CRS and ICANS was significantly greater in the innovative CAR-T group (91.3% and 30%, respectively) than in the conventional group (66.7% and 10%, respectively) (*p* = 0.044).

## 7. Incorporation of CAR-T Cell Therapy into Frontline Treatment of B-ALL

Prospective clinical trials are investigating the use of CAR-T for consolidation in patients with high-risk Ph-negative ALL, including those with a TP53 mutation, an IKZF1-plus signature, a Ph-like signature, and persistent MRD at the end of induction/consolidation [57]. A phase I/II study demonstrated the efficacy and safety of a memory-enriched anti-CD19 CAR-T in patients with R/R B-ALL [58]. This novel construct is being evaluated as a consolidation therapy for patients aged 55 years and older in first CR [59]. CAR T-cell therapy is also being investigated in the frontline setting of Ph-positive B-ALL [60].

## 8. T-Cell Acute Lymphoblastic Leukemia (T-ALL) and the Role of CAR-T Therapy

T-ALL predominantly affects adolescents and young adults and is slightly more common in males [61]. It has a poorer prognosis than B-ALL, partly due to higher rates of resistance and relapse [62]. Risk factors include a high white blood cell count at diagnosis and the presence of specific genetic abnormalities. NOTCH1 mutations are generally favorable, while complex karyotypes are adverse. MRD status post-induction is also a risk factor. Improved chemotherapy protocols have increased EFS rates, especially in pediatric patients (with rates of ~85–90% in some cohorts). Adult T-ALL has a less favorable prognosis, with a 5-year survival rate of around 40–50%. Traditional treatment consists of intensive chemotherapy, central nervous system prophylaxis, and a stem cell transplant for suitable candidates [63,64,65]. Challenges in T-ALL are related to antigen overlap. T-ALL cells express T-cell markers, such as CD3, CD7, and CD5, which complicates targeted immunotherapy.

CAR-T therapy targeting T-cell antigens is a promising frontier, especially for R/R cases, albeit with unique biological challenges such as fratricide. Current experimental therapies using genetically engineered, gene-edited CAR T-cells targeting CD7, CD5, and potentially multiple antigens show promising early results [66,67,68,69]. These innovations aim to treat R/R T-ALL with higher efficacy, safety, and durability, addressing the unique hurdles of targeting T-cell malignancies.

## 9. Toxicities Associated with CAR T

The most common toxicities are CRS and ICANS [70,71], managed primarily through early recognition, grading severity, and prompt treatment with tocilizumab and corticosteroids [72,73]. ICANS occurs in 20–60% of patients, with 12–30% experiencing severe symptoms. New European Society for Blood and Marrow Transplantation (EBMT) guidelines (2025) address non-classical neurological complications beyond traditional ICANS [74].

Preventive monitoring and adherence to evidence-based guidelines improve safety outcomes [75]. Tocilizumab has varied efficiency in CRS mitigation, with better results in diffuse large B-cell lymphoma compared to ALL. Management has improved with consensus definitions and guidelines facilitating recognition and timely intervention [76]. Early tocilizumab administration (within 24 h) is showing improved outcomes, with anakinra emerging as a second-line option for refractory cases [77]. Prophylactic or preemptive tocilizumab use is being investigated to reduce severe CRS risk without compromising antitumor efficacy. Beyond tocilizumab, corticosteroids play an important role in CRS management and are the mainstay of ICANS treatment.

A better understanding of pathophysiology reveals that disruption of brain microvascular endothelial cells leads to endothelial activation and neurotoxicity, with key cytokines including IL-10, IL-6, and IFN-γ [78]. Future directions for the treatment of toxicities include the development of predictive biomarkers for early intervention, CAR-T design modifications with safety switches, and novel therapeutic targets, including complement inhibition and neuroprotective strategies [79].

## 10. Expert Opinion

CAR-T is effective in treating R/R B-ALL [80]. Nevertheless, several challenges exist, such as relapse, severe toxicities, high costs, and limited accessibility. The future promises transformation through engineered CARs targeting multiple antigens, safer and longer-lasting T cells, universal off-the-shelf products, combination therapies, and sophisticated manufacturing. These innovations aim to improve the safety, efficacy, and accessibility of CAR T-cell therapy.

Relapse is a significant challenge. It occurs when leukemia cells lose or downregulate the target antigen, which makes them invisible to the engineered T-cells designed to attack that specific marker. This allows the cancer to evade immune detection and grow back, leading to disease relapse. Zebley et al. reported that CAR-T undergo rapid and widespread erasure of repressive DNA methylation programs at effector-associated genes [81]. CAR-T cell changes after infusion were characterized by the repression of genes associated with memory potential and a DNA methylation signature indicating a transition toward exhaustion-progenitor T cells. CD19-CAR-T cells underwent exhaustion-associated DNA methylation programming, suggesting that preventing this process could improve CAR T cell efficacy. Therefore, addressing antigen escape is paramount to enhancing long-term remission rates. After receiving CAR-T infusion, patients should undergo close monitoring for MRD using next-generation sequencing (NGS). Those who remain MRD-negative on serial assessments may be cured and may not require additional therapy. Those who demonstrate increasing MRD levels by NGS during follow-up after CAR-T are likely to experience relapse. This highlights the need for additional strategies, particularly allo-SCT. Modern trials should explore the role of CAR-T as a consolidation treatment for patients with high risk of relapse. Trials should also explore whether CAR-T can be combined with other treatments to improve outcomes. Innovation in engineering, combinations, and manufacturing will bring CAR-T closer to the front line, offering curative options to patients who previously had none. Multi-antigen targeting approaches aim to prevent relapse. Clinical trials show promising results, demonstrating that dual- or multispecific CAR-T can significantly improve response durability and reduce relapse rates. Next-generation, dual-targeting CARs are designed to enhance treatment efficacy and durability by overcoming antigen escape and tumor heterogeneity. These therapies represent a significant advancement in B-ALL immunotherapy (Table 5). Multi-target CAR-T strategies are essential for overcoming resistance in B-ALL by addressing tumor heterogeneity and antigen escape. Innovations such as dual- or multi-antigen recognition, logic-gated designs, and adaptable systems promise to enhance long-term remission and improve the durability of CARs.

**Figure 1:**
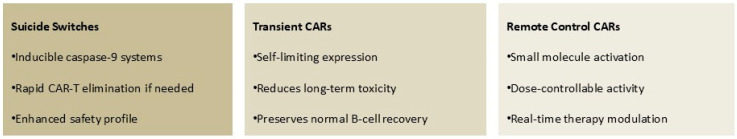
Technologies in development to prevent and manage side effects.

Another issue is to predict better, prevent, and manage short- and long-term side effects and gain a deeper understanding of the long-term impact of CAR-T on patients’ immune systems and overall health (Figure 1). Recent progress has focused on improving safety. Key strategies include switches and engineering CAR-T with built-in “kill switches” (e.g., inducible caspase-9), which allows clinicians to deactivate or eliminate cells if severe reactions occur; early intervention using cytokine blockers, such as tocilizumab and corticosteroids, at the first signs of CRS or ICANS; optimized dosing using stepwise or split dosing approaches to control immune activation; enhanced monitoring involving close, real-time assessments of cytokine levels and neurological status to help detect and treat side effects early. Other strategies include improving CARs’ design by developing CARs with modified signaling domains that produce fewer inflammatory cytokines and tailoring CAR-T therapy to individual patient risk factors to minimize adverse effects. To understand the full spectrum of effects over time, clinicians should design comprehensive, long-term follow-up studies on CAR-T recipients. Other ways to achieve this include identifying biomarkers that can predict the likelihood of long-term complications and investigating how genetic and epigenetic factors might influence long-term outcomes and susceptibility to side effects. Furthermore, clinicians should regularly assess patients’ quality of life to understand the therapy’s holistic long-term impact.

The future of CAR T-cell manufacturing aims to reduce production times from weeks to days through several ways, such as lower costs via automation and off-the-shelf products, using gene editing for universal, allogeneic CAR-T, standardizing processes to ensure high quality and consistency, and improving logistics with better cryopreservation and distributed manufacturing. These advancements will make CAR-T more accessible, affordable, and timely for patients worldwide. The industry is rapidly advancing toward more automated, scalable, and universal CAR T-cell manufacturing solutions. Key players lead in automation; others develop off-the-shelf products. CRISPR/TALEN tech makes universal options feasible. In this regard, a recent article published in the journal *Science Translational Medicine* outlines a potential future trajectory for CAR-T cell development [82]. The paper describes a phase I clinical trial designed to evaluate the safety of TT52CAR19 T cells, which are allogeneic CAR-T cells created using the CRISPR-Cas9 editing system. The researchers used an appropriate lentiviral vector to introduce molecular tools into T cells taken from healthy donors, silencing the TRAC gene and removing the gene that encodes the CD52 antigen. This genetic manipulation enables the cells to survive in the presence of the alemtuzumab antibody, thereby reducing the risk of GvHD. The resulting cells were cryopreserved and used in a phase I clinical trial involving six children with B-ALL who did not respond to standard treatments. In the latter case, the TALEN editing technique was used instead of CRISPR. This once again demonstrates the impact of this DNA manipulation technology in the field of therapeutic innovation. Thanks to genome editing techniques, these cells could be produced more easily and at a lower cost. Backed by efficacy and safety data, these advantages would make it easier to incorporate CAR-T cells into earlier lines of treatment. In vivo CAR programming is another method of genetically engineering a patient’s T cells to express CARs in their body [83]. This is achieved by delivering the CAR gene via vectors such as nanocarriers or viral vectors. This approach has several advantages over traditional ex vivo methods. It enables faster production and has the potential for broader applicability. It also eliminates the need to handle patient cells outside the body. Self-renewing CARs are a specific type of CAR T-cell therapy designed to function persistently within the body [84]. They are often modified to promote T-cell stemness and survival, but this can carry a risk of uncontrolled proliferation. Smart monitoring and response systems for CAR-T cells are advanced digital approaches that use AI and nanotechnology for the production and clinical monitoring of CAR-T therapies [85]. These systems aim to optimize the CAR-T cell manufacturing process, improve treatment durability and efficacy by overcoming immunosuppression, enable real-time monitoring of patient responses and toxicities, and enhance personalized care through AI-driven design and deployment. These cutting-edge innovations promise to make CAR T therapy more effective, safer, and more accessible in the near future.

The future of CAR-T in B-ALL should involve a clinical transformation in its use. This evolution involves shifting from a last-resort therapy to a first-line treatment, integrating it into upfront protocols for high-risk patients and achieving same-day availability. The timeline indicates that, by 2030, CAR-T therapy could transform B-ALL from a life-threatening condition into a highly curable disease with minimal long-term effects (Table 6). Key takeaways include a shift towards universal platforms for immediate availability, multi-target strategies to prevent resistance, and safety innovations that will make CAR-T therapy suitable for a broader range of patients, including those requiring first-line treatment.

## 11. Conclusions

These questions highlight the dynamic and evolving nature of B-ALL and CAR-T research. Ongoing studies and clinical trials are addressing many of these challenges to improve outcomes for patients with B-ALL and expand the applications of CAR T-cell therapy.

## Figures and Tables

**Table 1:** Chimeric Antigen Receptor T-cell process illustration in B acute lymphoblastic leukemia (ALL).

Main Process Steps:
T** **Cell** **Collection: Patient’s T cells are harvested via apheresis Genetic Modification: T cells are transduced with viral vectors carrying the CAR gene (typically anti-CD19 for B-ALL) Ex Vivo Expansion: Modified CAR-T cells are cultured and expanded to therapeutic numbers Reinfusion: Expanded CAR-T cells are infused back into the patient Mechanism of Action: Shows how CAR-T cells recognize and eliminate ALL cells
**Key Molecular Details:**
• CAR Structure: Single-chain variable fragment (scFv) for CD19 recognition, transmembrane domain, and intracellular signaling domains (CD3ζ and costimulatory domains like 4-1BB) • Target Recognition: Specific binding to CD19 antigen on B-ALL cells • Cytotoxic Mechanism: Release of perforin and granzymes leading to target cell apoptosis • Memory Formation: Development of long-term immune surveillance
**Clinical Context:**
• High efficacy rates: 80–90% complete remission in pediatric B-ALL • Side effects: Like cytokine release syndrome and the expected B-cell aplasia

**Table 2:** Approved CD19-directed CAR T-cell therapies in relapsed or refractory B acute lymphoblastic leukemia.

Agent	Costimulatory Molecule	CD19 Binding Domain	Indication
Tisagenlecleucel	4-1BB	FMC63 (murine scFv)	Patients aged ≤25 years
Brexucabtageneautoleucel	CD28	FMC63 (murine scFv)	Patients aged ≥25 years
Obecabtageneautoleucel	4-1BB	CAT (fast off-rate scFv)	Patients aged ≥18 years

**Table 3:** Consolidative allogeneic stem cell transplantation after CAR T in adult r/r B-ALL.

Author (Year)	Construct	Number of Patients	Age, Median, Range	Patients Who Responded and Proceeded to allo-SCT (% of Responders)	Median Time to allo-SCT Post CAR-T Infusion	TRM (%)	Main Results
Hay (2019 ) [40]	CD19.41BB	53	39 (20–76)	18/45 (40%)	70 (44–138)	23	With a median follow-up of 28.4 months after allo-SCT, the 2-year EFS and OS were 61% and 72%, respectively. Allo-SCT was associated with longer EFS compared with no allo-SCT.
Park (2018) [41]	19-28z CAR T	53	44 (23–74)	17/44 (39%)	74 (44–312)	35	After allo-SCT, median OS is 12.9 months. Among patients with a low disease burden, the median OS was 20.1 months.
Jiang (2019) [42]	CD19.41BB	58	28 (10–85)	21/47 (45%)	44 (33–89)	10	There was no difference in OS between MRD-CR patients who received allo-SCT and those who did not. However, EFS and RFS were significantly prolonged by allo-SCT in the subgroups.
Pan (2017) [43]	CD19 41BB	51		27/45 (60%)	84 (35–293)	7.5	Twenty-three of twenty-seven CR/CRi patients bridged to allo-SCT remained in MRD, with a median follow-up time of 206 (45–427) days.
Shah (2023) [44]	CD19.CD28	78	42.5 (18–84)	14/57 (25%)	95 (60–390)	Not reported	Patients with subsequent allo-SCT experienced favorable long-term response durability, with a median DOR of 44.2 months.
Aldoss (2024) [45]	CD19.various	45	31 (19–67)	45/45 26 (58%) and 19 (42%) received their first and second allo-SCT as consolidation post CAR-T therapy	93 (42–262)	2.4	With a median follow-up of 2.47 years (range: 0.13–6.93), 2-year OS, RFS, CIR, and NRM were 57.3%, 56.2%, 23.3%, and 20.4% respectively. Two-year OS, RFS, CIR, and NRM were not significantly different between patients who underwent their first vs. second transplant, respectively.
Roloff (2024) [46]	CD19.CD28	189	46 (18–81)	30/151 (20%)	99 (45–234)	17	In multivariable analysis, patients receiving consolidative allo-SCT (hazard ratio, 0.34 [95% CI, 0.14 to 0.85]) after CAR-T had superior PFS compared with those who did not receive any consolidation or maintenance therapy.
Shadman (2019) [47]	CD19.41BB	19	39 (23–74)	19/19 (100%)	72 (28–138)	21	At a median follow-up of 36 month, 1-year estimate of OS was 58% (95% CI, 40–85). Longer time from CAR-T therapy to allo-SCT (≥80 vs. <80 days) was associated with higher risk for death (hazard ratio [HR], 4.01; 95% CI, 1.14–14.0; *p* = 0.03) and higher NRM (HR, 4.4; 95% CI, 0.54–21.1; *p* = 0.19).
Roddie (2024) [36]	CD19.41BB	127	47 (20–81)	18/99 (18%)	101 (38–421)	Not reported	In 6 of 18 patients (33%), this procedure was a second allo-SCT. Of 11 patients who had persisting CAR T cells before allo-SCT and who had samples available afterward, none had CAR T cells detected after allo-SCT. No substantial difference in EFS or OS was observed between patients who received allo-SCT and those who did not.
Yang (2025) [48]	CD3ζ and 4-1BB		32.1 (15–67)	51 after achieving MRD-CR	2.6 (1.8–4.1)	6.7	This study provides the most extensive follow-up of real-world data on sequential allo-SCT after CAR-T therapy. Sequential allo-SCT after CAR-T treatment shows durable remissions in patients achieving MRD-negative CR. With 4 years of follow-up, OS reaches 68.9%, highlighting the long-term benefits. Sequential therapy demonstrates manageable safety, with an acute GVHD incidence of 31.4% and no GVHD-related deaths. Age and high-risk genetic factors are key determinants of long-term outcomes, requiring personalized treatment strategies.

allo-SCT = allogeneic stem-cell transplantation; EFS = event-free survival; OS = overall survival; RFS = relapse-free survival; CIR = cumulative incidence of relapse; NRM = non-relapse mortality; GVHD = graft-versus-host disease.

**Table 4:** Autologous vs. allogeneic CAR T-cell therapy.

Characteristic	Autologous CAR T-Cells	Allogeneic CAR T-Cells
Donor origin	Patient	Healthy donor
Production and manufacturing	Complex logistics	Scaled-up industrialized process
Cost	Currently high	Expected to be moderate
Main risks	CRS; CAR-related gene modifications; potential long-term adverse effects, such as B-cell aplasia	CRS; CAR and/or gene-editing-related gene modifications; GvHD; allogeneic cell rejection
Persistence	Months to years	Weeks to months
Redosing	Limited by cell number	Limited by the risk of alloimmunization

**Table 5:** Next-generation dual-targeting strategies.

Mechanism	Advantage	Status
**Bicistronic** **or tandem CAR designs targeting both CD19 and CD22**	Reduces antigen escape, maintains efficacy against CD19-negative variants	Phase I/II trials showing promising results with improved durability
**CRISPR/Cas9-engineered allogeneic T-cells with TCR/HLA knockout**	Off-the-shelf availability, reduced manufacturing time to hours	Multiple platforms in clinical development (UCART19, CTX110)
**CAR-T cells engineered to secrete supportive cytokines (IL-15, IL-12)**	Enhanced persistence and activity in immunosuppressive environments	Preclinical development with first trials expected in 2026
**Boolean logic circuits requiring multiple antigens for activation**	Improved specificity, reduced on-target off-tumor effects	Early preclinical development for enhanced safety

**Table 6:** Patient selection evolution.

Current Patient Selection (2025)	Future Patient Selection (2027–2030)	2030 Treatment Paradigm
• Relapsed/refractory B-ALL after ≥2 prior therapies • Chemotherapy-intolerant patients • Post-transplant relapse • High-risk genetics (e.g., Philadelphia chromosome-like) • Age: 3–25 years (tisagenlecleucel), 18+ (brexucabtagene)	• First-line therapy for high-risk patients • Minimal residual disease-positive patients • Prevention strategy post-chemotherapy • Expanded age ranges (infants to elderly) • Biomarker-guided selection (CAR-T readiness scores)	First-Line Integration CAR-T therapy integrated into upfront treatment protocols for high-risk B-ALL patients, replacing intensive chemotherapy phases
		Same-Day Treatment Universal CAR-T cells available immediately upon diagnosis, with personalized modifications made in real time
		Minimal Toxicity Smart safety switches and controlled activation systems eliminate severe CRS and neurotoxicity
		Durable Cures Self-renewing CAR-T cells provide lifelong surveillance and protection against relapse
